# (2*E*)-1-(2,5-Dimethyl­thio­phen-3-yl)-3-(3-nitro­phen­yl)prop-2-en-1-one

**DOI:** 10.1107/S1600536811047933

**Published:** 2011-11-16

**Authors:** Abdullah M. Asiri, Abdulrahman O. Al-Youbi, Salman A. Khan, M. Nawaz Tahir

**Affiliations:** a Department of Chemistry, Faculty of Science, King Abdulaziz University, Jeddah 21589, PO Box 80203, Saudi Arabia; b The Center of Excellence for Advanced Materials Reesrch, King Abdulaziz University, Jeddah 21589, PO Box 80203, Saudi Arabia; c Department of Physics, University of Sargodha, Sargodha, Pakistan

## Abstract

In the title compound, C_15_H_13_NO_3_S, the benzene ring and the five-membered heterocyclic ring are oriented at a dihedral angle of 12.00 (6)°. In the crystal, C—H⋯O inter­actions generate two types of cyclic motifs, *R*_2_^2^(14) and *R*_2_^2^(26), connecting the mol­ecules into tapes extending along [101]. In addition, there are π–π stacking inter­actions between the benzene and thio­phene rings with centroid-centroid distances of 3.7263 (14) and 3.7487 (14) Å.

## Related literature

For the synthesis of similar compounds, see: Asiri & Khan (2010 ▶, 2011 ▶); Kalirajan *et al.* (2009 ▶); Patil *et al.* (2009 ▶); Sarojini *et al.* (2006 ▶). For related structures and background references, see: Asiri *et al.* (2010*a* ▶,*b* ▶). For graph-set notation, see: Bernstein *et al.* (1995 ▶).
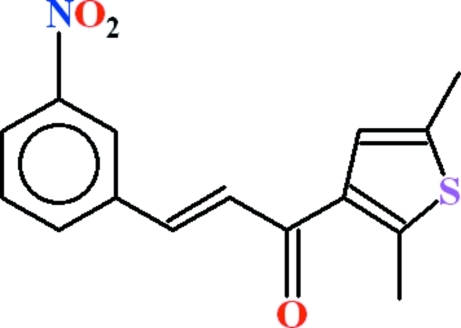


## Experimental

### 

#### Crystal data

C_15_H_13_NO_3_S
*M* *_r_* = 287.32
Monoclinic, 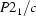

*a* = 7.3802 (5) Å
*b* = 13.7973 (9) Å
*c* = 13.4638 (8) Å
β = 96.997 (3)°
*V* = 1360.77 (15) Å^3^
*Z* = 4
Mo *K*α radiation
μ = 0.24 mm^−1^
*T* = 296 K
0.25 × 0.22 × 0.20 mm

#### Data collection

Bruker KAPPA APEXII CCD diffractometer
Absorption correction: multi-scan (*SADABS*; Bruker, 2005 ▶) *T*_min_ = 0.945, *T*_max_ = 0.955
10732 measured reflections
2466 independent reflections
1493 reflections with *I* > 2σ(*I*)
*R*_int_ = 0.051

#### Refinement

*R*[*F* ^2^ > 2σ(*F* ^2^)] = 0.047
*wR*(*F* ^2^) = 0.117
*S* = 1.03
2466 reflections
183 parameters
H-atom parameters constrained
Δρ_max_ = 0.17 e Å^−3^
Δρ_min_ = −0.25 e Å^−3^

### 

Data collection: *APEX2* (Bruker, 2009 ▶); cell refinement: *SAINT* (Bruker, 2009 ▶); data reduction: *SAINT*; program(s) used to solve structure: *SHELXS97* (Sheldrick, 2008 ▶); program(s) used to refine structure: *SHELXL97* (Sheldrick, 2008 ▶); molecular graphics: *ORTEP-3 for Windows* (Farrugia, 1997 ▶) and *PLATON* (Spek, 2009 ▶); software used to prepare material for publication: *WinGX* (Farrugia, 1999 ▶) and *PLATON*.

## Supplementary Material

Crystal structure: contains datablock(s) text, I. DOI: 10.1107/S1600536811047933/gk2432sup1.cif

Structure factors: contains datablock(s) I. DOI: 10.1107/S1600536811047933/gk2432Isup2.hkl

Supplementary material file. DOI: 10.1107/S1600536811047933/gk2432Isup3.cml

Additional supplementary materials:  crystallographic information; 3D view; checkCIF report

## Figures and Tables

**Table 1:** Hydrogen-bond geometry (Å, °)

*D*—H⋯*A*	*D*—H	H⋯*A*	*D*⋯*A*	*D*—H⋯*A*
C6—H6⋯O3^i^	0.93	2.46	3.373 (3)	168
C15—H15*B*⋯O2^ii^	0.96	2.59	3.339 (4)	135

Symmetry codes: (i) 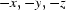; (ii) 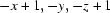.

**Fig. 1.**
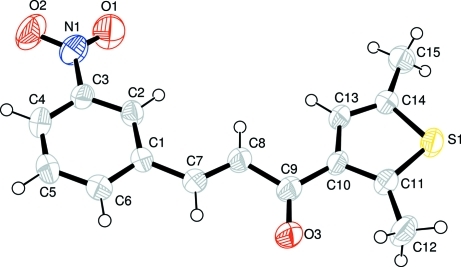
View of the title molecule with displacement ellipsoids drawn at the 50% probability level. H-atoms are shown as small spheres of arbitrary radii.

**Fig. 2.**
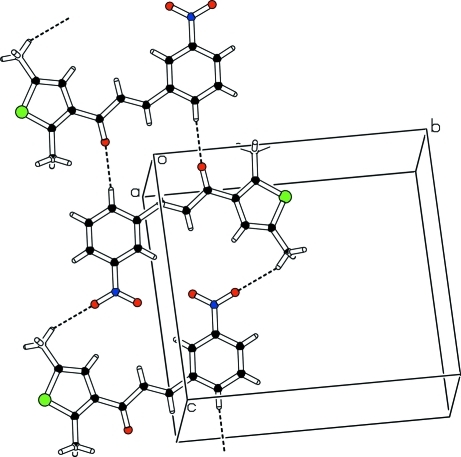
The partial packing (PLATON; Spek, 2009) showing the [1 0 1] tapes via R22(14) and R22(26) hydrogen-bond motifs.

## supplementary crystallographic information

### Comment

Claisen–Schmidt reaction is one of the most important reactions for the formation of α, β-unsaturated ketone by condensation between acetophenone and benzaldehyde (Asiri & Khan, 2010). The reaction is catalysed by bases, acids (Patil *et al.*, 2009). It is widely used in the synthesis of important intermediates (Asiri & Khan, 2011) or end-products, pharmaceuticals (Kalirajan *et al.*, 2009). It is also used in the field of matrial sciences such as photoelectronics, photophotonics, photodynamic therapy, electrochemical sensing, optical limiting, langmuir film and photoinitiated polymerization (Sarojini *et al.*, 2006). The title compound (I), (Fig. 1) has been synthesized as a pharmaceutical intermediate. Similar structures to (I) have been published earlier (Asiri *et al.*, 2010a,b and refereces therein).

In (I), the group A (C1—C6), the central propenone B (C7—C9/O3) and the group C (C10—C15/S1) are planar with r. m. s. deviation of 0.003, 0.012 and 0.008 Å, respectively. The dihedral angles between A/B, A/C and B/C are 9.88 (14), 12.00 (6) and 16.09 (12)°, respectively. The nitro group D (O1/N1/O2) is oriented at a dihedral angle of 8.4 (3)° with relation to the benzene ring A. The title compound consists of dimers due to intermolecular H-bonds of C—H···O type, where O-atom is of carbonyl and H-atom is of the nitrophenyl group. This H-bondings form a *R*_2_^2^(14) (Fig. 2) ring motif (Bernstein *et al.*, 1995). The same type of H-bonding between methyl and nitro groups consolidate the molecules in the form of one-dimensional polymers with *R*_2_^2^(26) ring motifs and extending along the [1 0 1] direction. Moreover there are π···π stacking interactions between the benzene and thiophene rings with centroid-centroid distances of 3.7263 (14)–3.7487 (14) Å.

### Experimental

A solution of 3-acetyl-2,5-dimethythiophene (0.38 g, 2.5 mmol) and 3-nitro-benzaldehyde (0.37 g, 2.5 mmol) in ethanolic solution of NaOH (3.0 g in 10 ml of methanol) was stirred for 16 h at room temperature. The solution was poured into ice cold water of pH=2 (pH adjusted by HCl). The solid separated was filtered and crystallized from methanol:chloroform to affoard light yellow prisms of (I).

Yield: 78%; m.p. 403–404 K.

IR (KBr) ν_max_ cm^-1^: 3012 (Ar—H), 2926 (C—H), 1628 (C═O), 1568 (C═C).

1H NMR (DMSO-d_6_) (δ/p.p.m.): 8.47 (d, J = 1.8 Hz), 8.23 (d, J = 1.2 Hz), 7.73 (d, C═CH, J = 15.6 Hz), 7.40 (d, CH═C, J = 15.6 Hz), 7.89(d, J=7.2 Hz), 7.61 (d, J = 7.8 Hz), 7.27 (s, Ar—H), 2.72 (s, CH_3_), 2.39 (s, CH_3_).

### Refinement

The H atoms were positioned geometrically (C–H = 0.93–0.96 Å) and refined as riding with *U*_iso_(H) = x*U*_eq_(C), where x = 1.5 for methyl and x = 1.2 for aryl H-atoms.

### Crystal data

**Table:**

C_15_H_13_NO_3_S	*F*(000) = 600
*M**_r_* = 287.32	*D*_x_ = 1.402 Mg m^−^^3^
Monoclinic, *P*2_1_/*c*	Mo *K*α radiation, λ = 0.71073 Å
Hall symbol: -P 2ybc	Cell parameters from 1493 reflections
*a* = 7.3802 (5) Å	θ = 2.1–25.3°
*b* = 13.7973 (9) Å	µ = 0.24 mm^−^^1^
*c* = 13.4638 (8) Å	*T* = 296 K
β = 96.997 (3)°	Prism, yellow
*V* = 1360.77 (15) Å^3^	0.25 × 0.22 × 0.20 mm
*Z* = 4	

### Data collection

**Table:**

Bruker KAPPA APEXII CCD diffractometer	2466 independent reflections
Radiation source: fine-focus sealed tube	1493 reflections with *I* > 2σ(*I*)
graphite	*R*_int_ = 0.051
Detector resolution: 8.10 pixels mm^-1^	θ_max_ = 25.3°, θ_min_ = 2.1°
ω scans	*h* = −8→8
Absorption correction: multi-scan (*SADABS*; Bruker, 2005)	*k* = −13→16
*T*_min_ = 0.945, *T*_max_ = 0.955	*l* = −16→16
10732 measured reflections	

### Refinement

**Table:**

Refinement on *F*^2^	Primary atom site location: structure-invariant direct methods
Least-squares matrix: full	Secondary atom site location: difference Fourier map
*R*[*F*^2^ > 2σ(*F*^2^)] = 0.047	Hydrogen site location: inferred from neighbouring sites
*wR*(*F*^2^) = 0.117	H-atom parameters constrained
*S* = 1.03	*w* = 1/[σ^2^(*F*_o_^2^) + (0.0498*P*)^2^ + 0.0228*P*] where *P* = (*F*_o_^2^ + 2*F*_c_^2^)/3
2466 reflections	(Δ/σ)_max_ < 0.001
183 parameters	Δρ_max_ = 0.17 e Å^−^^3^
0 restraints	Δρ_min_ = −0.25 e Å^−^^3^

### Special details

**Table:**

Geometry. Bond distances, angles *etc*. have been calculated using the rounded fractional coordinates. All su's are estimated from the variances of the (full) variance-covariance matrix. The cell e.s.d.'s are taken into account in the estimation of distances, angles and torsion angles
Refinement. Refinement of *F*^2^ against ALL reflections. The weighted *R*-factor *wR* and goodness of fit *S* are based on *F*^2^, conventional *R*-factors *R* are based on *F*, with *F* set to zero for negative *F*^2^. The threshold expression of *F*^2^ > σ(*F*^2^) is used only for calculating *R*-factors(gt) *etc*. and is not relevant to the choice of reflections for refinement. *R*-factors based on *F*^2^ are statistically about twice as large as those based on *F*, and *R*- factors based on ALL data will be even larger.

### Fractional atomic coordinates and isotropic or equivalent isotropic displacement parameters (Å^2^)

**Table:**

	*x*	*y*	*z*	*U*_iso_*/*U*_eq_	
S1	0.21173 (10)	0.46796 (5)	0.18440 (5)	0.0520 (3)	
O1	0.4422 (3)	−0.10573 (18)	0.51656 (15)	0.0876 (10)	
O2	0.5017 (3)	−0.25617 (17)	0.49723 (15)	0.0892 (10)	
O3	0.0404 (3)	0.17111 (14)	0.06130 (14)	0.0744 (8)	
N1	0.4392 (3)	−0.1786 (2)	0.46575 (18)	0.0607 (10)	
C1	0.2331 (3)	−0.07879 (19)	0.22025 (18)	0.0413 (9)	
C2	0.3105 (3)	−0.08450 (19)	0.32014 (17)	0.0431 (9)	
C3	0.3587 (3)	−0.17332 (19)	0.36030 (18)	0.0444 (9)	
C4	0.3343 (4)	−0.2574 (2)	0.3066 (2)	0.0577 (11)	
C5	0.2586 (4)	−0.2528 (2)	0.2088 (2)	0.0618 (11)	
C6	0.2074 (4)	−0.1642 (2)	0.1663 (2)	0.0530 (10)	
C7	0.1775 (3)	0.01281 (19)	0.17143 (19)	0.0457 (10)	
C8	0.2028 (4)	0.10257 (19)	0.20395 (18)	0.0490 (10)	
C9	0.1319 (4)	0.1860 (2)	0.14180 (18)	0.0491 (10)	
C10	0.1748 (3)	0.28475 (18)	0.17826 (18)	0.0419 (9)	
C11	0.1514 (3)	0.36459 (18)	0.11758 (18)	0.0438 (9)	
C12	0.0882 (4)	0.3718 (2)	0.00750 (18)	0.0616 (11)	
C13	0.2414 (3)	0.31061 (19)	0.27906 (17)	0.0439 (9)	
C14	0.2659 (3)	0.40587 (19)	0.29503 (17)	0.0433 (9)	
C15	0.3299 (4)	0.4582 (2)	0.39024 (19)	0.0597 (11)	
H2	0.32910	−0.02870	0.35879	0.0517*	
H4	0.36845	−0.31670	0.33608	0.0691*	
H5	0.24165	−0.30911	0.17094	0.0742*	
H6	0.15459	−0.16188	0.10004	0.0636*	
H7	0.11464	0.00698	0.10753	0.0548*	
H8	0.26607	0.11362	0.26696	0.0588*	
H12A	−0.04283	0.37068	−0.00322	0.0925*	
H12B	0.13538	0.31800	−0.02658	0.0925*	
H12C	0.13152	0.43128	−0.01806	0.0925*	
H13	0.26568	0.26483	0.32962	0.0527*	
H15A	0.45201	0.48132	0.38808	0.0894*	
H15B	0.32800	0.41479	0.44581	0.0894*	
H15C	0.25052	0.51215	0.39790	0.0894*	

### Atomic displacement parameters (Å^2^)

**Table:**

	*U*^11^	*U*^22^	*U*^33^	*U*^12^	*U*^13^	*U*^23^
S1	0.0670 (5)	0.0373 (4)	0.0511 (4)	0.0052 (4)	0.0043 (3)	0.0067 (3)
O1	0.132 (2)	0.0787 (18)	0.0482 (13)	0.0074 (15)	−0.0048 (13)	−0.0029 (13)
O2	0.120 (2)	0.0730 (16)	0.0699 (16)	0.0207 (15)	−0.0078 (14)	0.0294 (13)
O3	0.1077 (17)	0.0512 (13)	0.0544 (12)	−0.0006 (12)	−0.0302 (12)	0.0031 (10)
N1	0.0710 (17)	0.0627 (19)	0.0483 (16)	0.0049 (15)	0.0073 (12)	0.0161 (15)
C1	0.0439 (16)	0.0344 (15)	0.0453 (15)	−0.0035 (13)	0.0037 (12)	0.0001 (13)
C2	0.0515 (17)	0.0361 (16)	0.0420 (15)	0.0013 (13)	0.0067 (12)	−0.0021 (13)
C3	0.0471 (17)	0.0411 (17)	0.0445 (15)	−0.0004 (14)	0.0036 (12)	0.0072 (14)
C4	0.067 (2)	0.0354 (17)	0.069 (2)	0.0034 (15)	0.0018 (16)	0.0096 (16)
C5	0.079 (2)	0.0352 (17)	0.068 (2)	−0.0013 (16)	−0.0044 (17)	−0.0073 (15)
C6	0.0605 (19)	0.0453 (18)	0.0500 (16)	−0.0025 (15)	−0.0066 (14)	−0.0031 (15)
C7	0.0500 (17)	0.0423 (18)	0.0429 (16)	−0.0023 (14)	−0.0016 (12)	0.0036 (13)
C8	0.0639 (19)	0.0420 (18)	0.0384 (15)	0.0004 (14)	−0.0047 (13)	0.0029 (13)
C9	0.0582 (18)	0.0476 (18)	0.0396 (15)	0.0023 (14)	−0.0015 (14)	0.0062 (14)
C10	0.0518 (17)	0.0342 (16)	0.0382 (14)	0.0054 (12)	−0.0003 (12)	0.0064 (12)
C11	0.0500 (17)	0.0415 (16)	0.0396 (14)	0.0085 (13)	0.0038 (12)	0.0040 (13)
C12	0.084 (2)	0.0536 (19)	0.0449 (16)	0.0124 (16)	−0.0011 (15)	0.0121 (14)
C13	0.0527 (17)	0.0402 (17)	0.0376 (15)	0.0000 (13)	0.0006 (12)	0.0106 (12)
C14	0.0451 (16)	0.0408 (17)	0.0432 (15)	0.0011 (13)	0.0023 (12)	0.0023 (13)
C15	0.073 (2)	0.0519 (19)	0.0524 (17)	−0.0011 (16)	0.0008 (15)	−0.0062 (14)

### Geometric parameters (Å, °)

**Table:**

S1—C11	1.716 (3)	C10—C13	1.431 (3)
S1—C14	1.723 (2)	C11—C12	1.502 (3)
O1—N1	1.215 (4)	C13—C14	1.341 (4)
O2—N1	1.221 (4)	C14—C15	1.497 (4)
O3—C9	1.223 (3)	C2—H2	0.9300
N1—C3	1.473 (3)	C4—H4	0.9300
C1—C2	1.398 (3)	C5—H5	0.9300
C1—C6	1.385 (4)	C6—H6	0.9300
C1—C7	1.460 (4)	C7—H7	0.9300
C2—C3	1.369 (4)	C8—H8	0.9300
C3—C4	1.367 (4)	C12—H12A	0.9600
C4—C5	1.368 (4)	C12—H12B	0.9600
C5—C6	1.383 (4)	C12—H12C	0.9600
C7—C8	1.319 (4)	C13—H13	0.9300
C8—C9	1.481 (4)	C15—H15A	0.9600
C9—C10	1.470 (4)	C15—H15B	0.9600
C10—C11	1.370 (3)	C15—H15C	0.9600
			
C11—S1—C14	93.33 (12)	C13—C14—C15	129.3 (2)
O1—N1—O2	123.4 (2)	C1—C2—H2	120.00
O1—N1—C3	118.7 (2)	C3—C2—H2	120.00
O2—N1—C3	118.0 (2)	C3—C4—H4	121.00
C2—C1—C6	118.1 (2)	C5—C4—H4	121.00
C2—C1—C7	122.8 (2)	C4—C5—H5	120.00
C6—C1—C7	119.2 (2)	C6—C5—H5	120.00
C1—C2—C3	119.1 (2)	C1—C6—H6	119.00
N1—C3—C2	118.7 (2)	C5—C6—H6	119.00
N1—C3—C4	118.7 (2)	C1—C7—H7	115.00
C2—C3—C4	122.7 (2)	C8—C7—H7	115.00
C3—C4—C5	118.8 (3)	C7—C8—H8	119.00
C4—C5—C6	119.9 (3)	C9—C8—H8	119.00
C1—C6—C5	121.5 (2)	C11—C12—H12A	109.00
C1—C7—C8	130.0 (2)	C11—C12—H12B	109.00
C7—C8—C9	121.1 (2)	C11—C12—H12C	109.00
O3—C9—C8	119.3 (2)	H12A—C12—H12B	109.00
O3—C9—C10	121.7 (2)	H12A—C12—H12C	109.00
C8—C9—C10	119.0 (2)	H12B—C12—H12C	109.00
C9—C10—C11	122.7 (2)	C10—C13—H13	123.00
C9—C10—C13	125.7 (2)	C14—C13—H13	123.00
C11—C10—C13	111.7 (2)	C14—C15—H15A	110.00
S1—C11—C10	110.48 (18)	C14—C15—H15B	109.00
S1—C11—C12	119.47 (19)	C14—C15—H15C	109.00
C10—C11—C12	130.0 (2)	H15A—C15—H15B	110.00
C10—C13—C14	114.9 (2)	H15A—C15—H15C	109.00
S1—C14—C13	109.66 (18)	H15B—C15—H15C	109.00
S1—C14—C15	121.10 (19)		
			
C14—S1—C11—C10	−0.85 (19)	C3—C4—C5—C6	−0.4 (4)
C14—S1—C11—C12	−179.6 (2)	C4—C5—C6—C1	0.9 (4)
C11—S1—C14—C13	1.22 (19)	C1—C7—C8—C9	−179.2 (2)
C11—S1—C14—C15	−178.9 (2)	C7—C8—C9—O3	3.8 (4)
O1—N1—C3—C2	7.8 (3)	C7—C8—C9—C10	−175.7 (2)
O1—N1—C3—C4	−171.9 (2)	O3—C9—C10—C11	−14.6 (4)
O2—N1—C3—C2	−171.3 (2)	O3—C9—C10—C13	164.2 (3)
O2—N1—C3—C4	9.0 (3)	C8—C9—C10—C11	164.9 (2)
C6—C1—C2—C3	0.5 (3)	C8—C9—C10—C13	−16.3 (4)
C7—C1—C2—C3	179.9 (2)	C9—C10—C11—S1	179.2 (2)
C2—C1—C6—C5	−0.9 (4)	C9—C10—C11—C12	−2.3 (4)
C7—C1—C6—C5	179.6 (2)	C13—C10—C11—S1	0.3 (2)
C2—C1—C7—C8	7.4 (4)	C13—C10—C11—C12	178.8 (2)
C6—C1—C7—C8	−173.2 (3)	C9—C10—C13—C14	−178.2 (2)
C1—C2—C3—N1	−179.7 (2)	C11—C10—C13—C14	0.7 (3)
C1—C2—C3—C4	0.0 (4)	C10—C13—C14—S1	−1.3 (3)
N1—C3—C4—C5	179.6 (2)	C10—C13—C14—C15	178.9 (2)
C2—C3—C4—C5	0.0 (4)		

### Hydrogen-bond geometry (Å, °)

**Table:**

*D*—H···*A*	*D*—H	H···*A*	*D*···*A*	*D*—H···*A*
C6—H6···O3^i^	0.93	2.46	3.373 (3)	168
C15—H15B···O2^ii^	0.96	2.59	3.339 (4)	135

Symmetry codes: (i) −*x*, −*y*, −*z*; (ii) −*x*+1, −*y*, −*z*+1.
